# Uterine artery pseudoaneurysm requiring embolization in pregnancy: a case report and review of the literature

**DOI:** 10.1186/s42155-018-0040-2

**Published:** 2018-11-21

**Authors:** Lilian Ugwumadu, Kevin Hayes, Anna-Maria Belli, Susan Heenan, Ian Loftus

**Affiliations:** 1 Department of Obstetrics & Gynaecology, St George’s University Hospitals NHS Foundation Trust, London, UK; 2 Department of Radiology, St George’s University Hospitals NHS Foundation Trust, London, UK; 3 Department of Vascular Surgery, St George’s University Hospitals NHS Foundation Trust, London, UK

**Keywords:** Pseudoaneurysm, Uterine artery Pseudoaneurysm, Embolization

## Abstract

**Background:**

Uterine Artery Pseudoaneurysm is a rare cause of pelvic pain and haemorrhage in pregnancy. It should be considered in the differential diagnosis of pregnant women presenting with abdominal pain and is readily diagnosed by colour Doppler ultrasound. If left untreated, they may bleed into the peritoneum causing severe pain and haemorrhagic shock and may progress to maternal and fetal death.

**Case presentation:**

We describe a case of a woman presenting with severe right iliac fossa pain at 26 weeks gestation attributed to a right uterine artery pseudoaneurysm diagnosed on duplex ultrasound which was successfully treated by uterine artery embolization at 28 weeks gestation without complication to the fetus.

**Conclusion:**

Uterine artery embolization appears to be a safe and effective method to treat pseudoaneurysm during pregnancy without compromising uteroplacental perfusion.

## Background

Uterine artery pseudoaneurysm is a very rare cause of blood loss during pregnancy. It occurs after vascular damage when blood flows through the arterial wall layers, creating a pseudoaneurysm (Zimon et al. 1999). With improvements in imaging technology, uterine artery pseuoaneurysms are diagnosed with increasing frequency. Previously, the majority of uterine artery aneurysms were treated by laparotomy and internal iliac artery ligation (Descargues et al. 2001). In recent years, uterine artery embolization has become the accepted and reliable method to treat uterine artery pseudoaneurysm in haemodynamically stable patients (Kwon and Kim 2002). We present a case of pseudoaneurysm of the uterine artery presenting in pregnancy, diagnosed using duplex Doppler sonography which was successfully treated by uterine artery embolization and a review of the literature.

## Case presentation

**Fig. 1:**
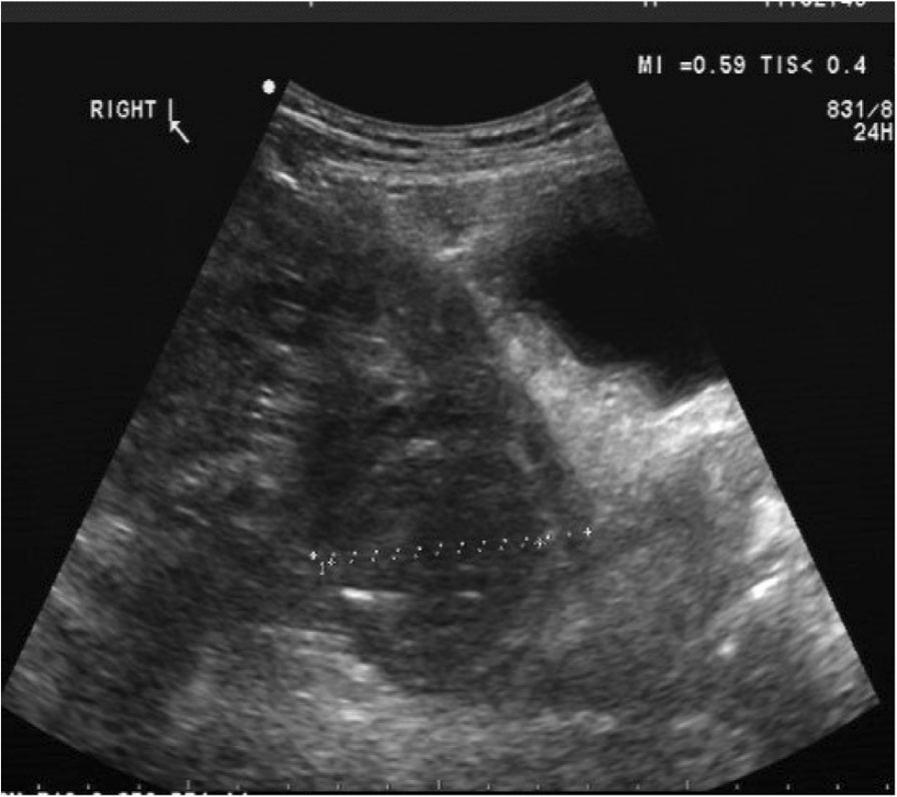
Transvaginal ultrasound image of the right uterine artery pseudoaneuyrsm

**Fig. 2:**
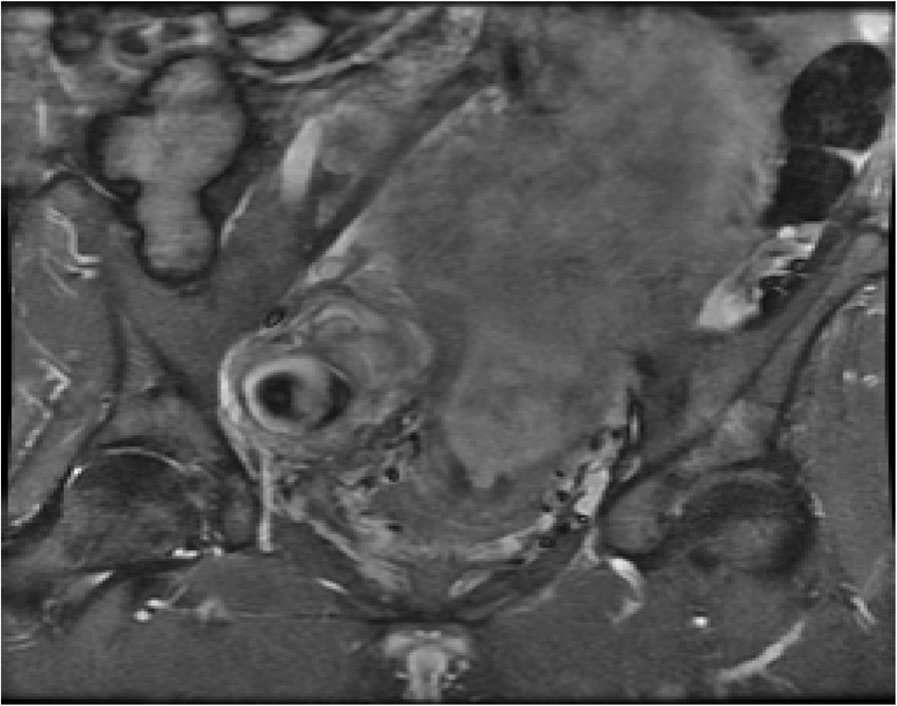
MRI: right uterine artery pseudoaneurysm

**Fig. 3:**
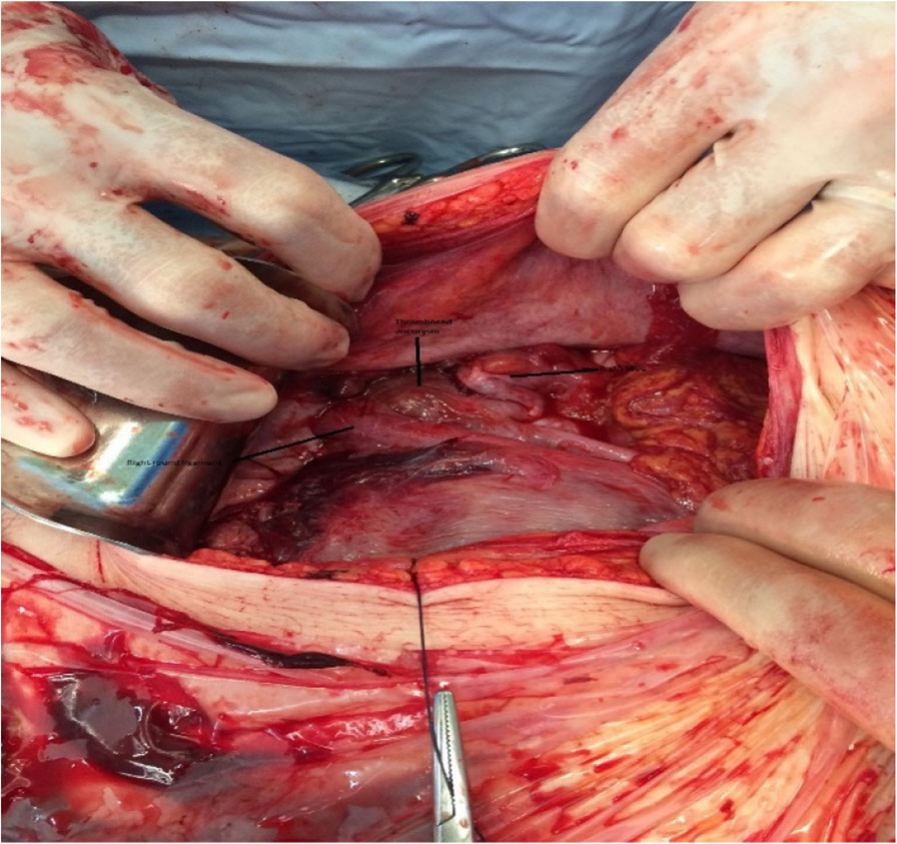
Thrombosed pseudoaneuyrsm seen at Caesarean section

**Fig. 4:**
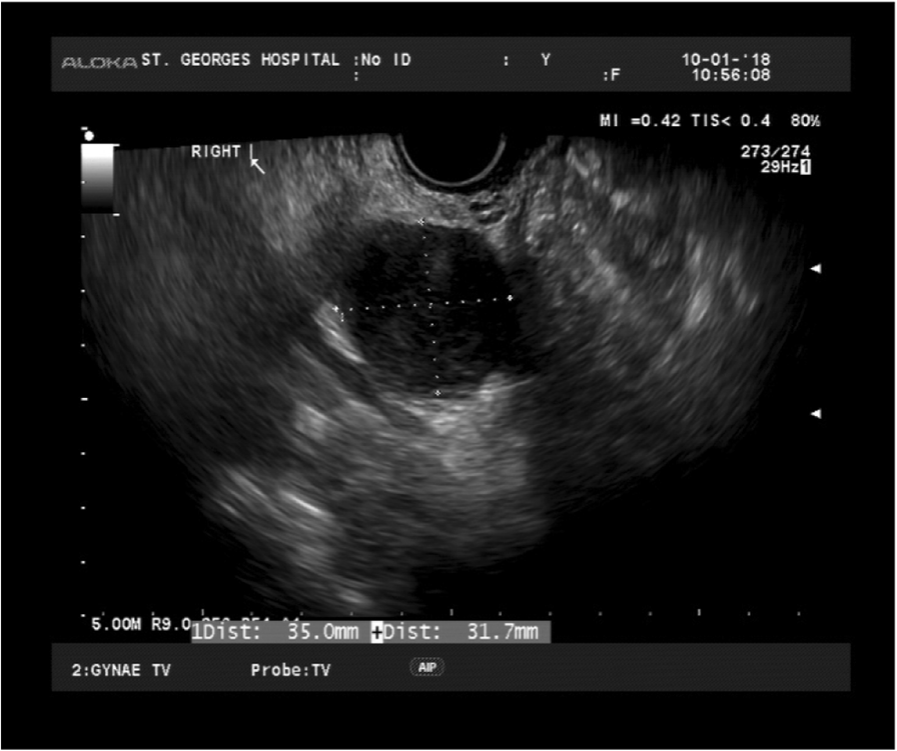
Transvaginal ultrasound image of the thrombosed right uterine artery pseudoaneuyrsm

A 38-year-old gravida 1, para 0 was transferred to our unit at 26 weeks of gestation with severe right iliac fossa pain and a suspicion of threatened preterm labour. She presented with a 3 day history of right iliac fossa pain, nausea, vomiting and diarrhoea. Vaginal examination and her fetal fibronectin test was negative. However, transabdominal and transvaginal ultrasound examination revealed a 6.0 × 5.5 × 5.9 cm vascular mass within the right pelvis with a clear arterial feeder and turbulent swirling intraluminal flow, leading to the diagnosis of a pseudoaneurysm (Fig. 1). The diagnosis was subsequently confirmed by Magnetic resonance imaging (MRI) (Fig. 2). The patient was discussed in our multidisciplinary team meeting with interventional radiologists, vascular surgeons, and anaesthetists. The risk of imminent rupture was assessed as high due to the severity of her symptoms and a 5–10 mm overall increase in the size of the pseudoaneurysm documented by sequential MRI 14 days apart. Surgery was considered high risk and so selective embolization of the pseudoaneurysm was performed. This was performed under local anaesthetic in the Interventional Radiology suite with full preparation for emergency delivery by the obstetric and anaesthetic team in case of fetal distress. The right uterine artery was identified angiographically from a contralateral femoral arterial puncture and selectively catheterised using a microcatheter. The artery was small but extravasation into the presumed pseudoaneurysm was identified near its proximal portion. The artery was embolised with a series of microcoils across the neck of the pseudoaneurysm to block flow. The procedure was uneventful and the fetus showed a continuous reactive heart rate pattern. Follow up ultrasound showed a completely thrombosed pseudoaneurysm with no flow. She had regular follow ups in the antenatal clinic with no sign of recurrence. A planned caesarean section was performed at 38 weeks gestation. A healthy baby boy was born weighing 2696 g with a blood loss of 1100mls. During the caesarean section, the thrombosed pseudoaneurysm was seen below the right broad ligament and all other pelvic organs looked completely normal (Fig. 3). An ultrasound scan 3 months postpartum showed a small completely thrombosed pseudoaneurysm (Fig. 4).

## Discussion

Pseudoaneurysms arise from vascular damage leading to a disruption and defect within the arterial wall. When the damaged artery does not seal properly, blood escapes, dissects the adjacent tissues around the damaged artery and forms a perfused sac that communicates with the arterial lumen (Zimon et al. 1999). Pseudoaneurysm of the uterine artery has been described as a consequence of Caesarean section (Descargues et al. 2001), dilatation and curettage (Kwon and Kim 2002), hysterectomy (Lee et al. 2001), myomectomy (Higon et al. 2007), and after an uncomplicated vaginal delivery (McGonegle et al. 2006). Our patient had no known risk factor for its occurrence and the aetiology remains unknown.

Although the effect of pregnancy on pseudoaneurysm is unknown, due to their rarity, pregnancy has been associated with an increased risk of rupture of true aneurysms, particularly during the third trimester and puerperium (Barrett et al. 1982). It is believed that the hormonal and haemodynamic environment of pregnancy causes changes in arterial content and organization that weaken arterial walls and predispose to aneurysmal rupture (Barrett et al. 1982). Pseudoaneurysms may be asymptomatic and detected only incidentally during radiologic investigation of other conditions or during surgery (McDermott et al. 1994). Symptomatic pseudoaneurysms manifest with severe vaginal bleeding or abdominal pain, with rupture the most serious cause of morbidity and mortality (Osol and Mandala 2009). Mortality has been described, after post-mortem, due to a case of ruptured pseudoaneurysm in pregnancy (Cardia et al. 2009). Non-invasive radiological imaging techniques such as ultrasound, CT and MR imaging facilitate the diagnosis of uterine artery pseudoaneurysm but ultrasound and MRI are safer in pregnancy (Belli et al. 2012). Smaller pseudoaneurysm of less than 10 mm may be difficult to identify on ultrasound and will require other forms of imaging such as MRI. There have been no documented teratogenic effects after the inadvertent administration of MR imaging contrast agents in pregnant women but it should only be used when additional information or treatment outweighs the potential risks (Tremblay et al. 2012).

Treatment options have evolved over the past few years from open surgical management to less invasive image guided interventions contributing to a dramatic decline in morbidity and mortality rates. Previously, the majority of uterine artery aneurysms were treated by laparotomy and internal iliac artery ligation (Descargues et al. 2001). In recent years, image guided catheter embolization has become the accepted and reliable method to treat uterine artery pseudoaneurysm in haemodynamically stable patients (Kwon and Kim 2002). There are other possible methods of treatment which could be considered such as US guided thrombin injection (Hong et al. 2012) or a covered stent (Jesinger et al. 2013) depending on the expertise of the operator and local experience.

Three cases of uterine artery aneurysms during pregnancy requiring selective embolization are reported in literature (Laubach et al. 2000; Cornette et al. 2014; Maignien et al. 2015) but this is the second case of successful diagnosis and treatment of uterine artery pseudo-aneurysm during pregnancy resulting in a term delivery.

These case reports suggest that fetuses can tolerate selective unilateral uterine artery embolization and though only two, were important pieces of literature in our decision making for active intervention. Uterine blood flow should not be sacrificed routinely but it can be done safely in a potentially life threatening condition as demonstrated by our case report and others in the literature. Blood supply from collaterals and the contralateral uterine artery allowed the pregnancy to continue safely. Additionally, there is evidence that the uterine artery frequently recanalises after embolisation (Das et al. 2013).

It is essential the fetus is monitored during and immediately after the procedure and regular fetal growth and placental perfusion assessments are maintained throughout the pregnancy. We were reassured by her assessments which showed the fetus was growing acceptably without compromise to placental perfusion. We considered a vaginal delivery but opted for an elective caesarean section, because of the uncertainty of the effects of uterine contractions on the thrombosed pseudoaneurysm and patient choice. The choice of timing of delivery was due to the difficult pregnancy, some continued pain and to minimise the chance of spontaneous labour.

## Conclusion

In summary, uterine artery pseudoaneurysm should be considered in women with severe abdominal pain in pregnancy. It is a rare but potentially life threatening condition for the mother and fetus. Duplex Ultrasound is the most useful tool in its diagnosis in pregnancy. Management with selective unilateral uterine artery embolization appears to be safe, tolerated by the fetus and helpful in prolonging the pregnancy safely and reducing the morbidity associated with preterm birth.

## Abbreviations

CT: Computed tomography
MRI: Magnetic resonance imaging
